# Unlocking the Genetic Diversity of Maize Landraces with Doubled Haploids Opens New Avenues for Breeding

**DOI:** 10.1371/journal.pone.0057234

**Published:** 2013-02-22

**Authors:** Alexander Strigens, Wolfgang Schipprack, Jochen C. Reif, Albrecht E. Melchinger

**Affiliations:** 1 Institute of Plant Breeding, Seed Science and Population Genetics, University of Hohenheim, Stuttgart, Germany; 2 State Plant Breeding Institute, University of Hohenheim, Stuttgart, Germany; United States Department of Agriculture, United States of America

## Abstract

Landraces are valuable genetic resources for broadening the genetic base of elite germplasm in maize. Extensive exploitation of landraces has been hampered by their genetic heterogeneity and heavy genetic load. These limitations may be overcome by the *in-vivo* doubled haploid (DH) technique. A set of 132 DH lines derived from three European landraces and 106 elite flint (EF) lines were genotyped for 56,110 single nucleotide polymorphism (SNP) markers and evaluated in field trials at five locations in Germany in 2010 for several agronomic traits. In addition, the landraces were compared with synthetic populations produced by intermating DH lines derived from the respective landrace. Our objectives were to (1) evaluate the phenotypic and molecular diversity captured within DH lines derived from European landraces, (2) assess the breeding potential (usefulness) of DH lines derived from landraces to broaden the genetic base of the EF germplasm, and (3) compare the performance of each landrace with the synthetic population produced from the respective DH lines. Large genotypic variances among DH lines derived from landraces allowed the identification of DH lines with grain yields comparable to those of EF lines. Selected DH lines may thus be introgressed into elite germplasm without impairing its yield level. Large genetic distances of the DH lines to the EF lines demonstrated the potential of DH lines derived from landraces to broaden the genetic base of the EF germplasm. The comparison of landraces with their respective synthetic population showed no yield improvement and no reduction of phenotypic diversity. Owing to the low population structure and rapid decrease of linkage disequilibrium within populations of DH lines derived from landraces, these would be an ideal tool for association mapping. Altogether, the DH technology opens new opportunities for characterizing and utilizing the genetic diversity present in gene bank accessions of maize.

## Introduction

According to molecular evidence, maize (*Zea mays* L.) was introduced into Europe over two distinct paths. A first introduction of Caribbean Flint maize in Spain by Colombus in 1493 was followed by introductions of Northern Flints from North America to North-Western Europe during the 16^th^ century [1]. This flint maize was cultivated as open-pollinated populations in different regions of Europe over centuries, resulting in a broad diversity of landraces. Natural selection promoted adaptation to the cool and wet climatic conditions prevailing in large parts of the continent [2], while artificial selection through saving desirable ears for the next growing season shaped the landraces according to farmers' preferences. Hybridization of flint populations from Spain with those from the northern introduction in the Pyrenean region produced a novel germplasm that was most likely the cradle of the European elite flint (EF) germplasm [3]. Despite good adaptation of landraces to local climatic and soil conditions, the advent of highly productive hybrids in the 1960s resulted in a rapid decline of their cultivation [4]. Fortunately, the value of landraces as genetic resources was recognized before their extinction. They were collected at their growing locations and are being conserved *ex situ*, in gene banks.

During the early cycles of hybrid breeding in the 1950's and 1960's, a portion of the genetic diversity and specific adaptation of the European landraces was captured in the founder lines of the modern European flint heterotic pool [5]. However, only few inbred lines derived from a few landraces contributed to the elite breeding material. Broadening the genetic base of the elite breeding pool through introgression of additional material from European landraces might, therefore, be of great interest [6]. Extensive characterization studies of landraces for morphological traits [10], [11], early growth and cold tolerance [12]–[16], adaptation to low nitrogen fertilization [17], [18], and pest resistance [19], [20], often identified landraces with desirable traits. Germplasm collections of European landraces were also extensively genotyped with molecular markers to determine their origin, relatedness, and degree of genetic diversity [1], [7]–[9], which revealed their broad genetic diversity and potential for further mining of favorable alleles. Nevertheless, the presence of deleterious and recessive lethal alleles in the landraces, commonly referred to as genetic load or genetic burden, has so far hampered their direct use in breeding [21].

Inbreeding uncovers the genetic load and can be used to purge landraces from lethal or detrimental alleles [22]. However, line development by recurrent selfing is extremely cumbersome. Recessive lethal alleles masked in heterozygous plants during early generations result in losses of lines in advanced selfing generations, due to increasing homozygosity. Use of the *in-vivo* doubled haploid (DH) technique [23] was proposed to overcome these drawbacks [7]. This technique is routinely used for line development in commercial breeding programs in Europe and the US and could offer several advantages with respect to the exploitation of landraces. First, recessive lethal alleles are expressed already at the haploid stage, which should reduce a part of the genetic load at the very beginning of line development [24], [25]. Second, alleles present in heterogeneous populations are fixed in a single step in homozygous lines, ideally capturing most of the variation present in the original source [26]. Third, DH lines can be reproduced *ad libitum* and evaluated with any desired degree of precision in replicated trials, whereas landraces in any generation represent a conglomerate of highly diverse, unique and non-reproducible individuals. This enables maintenance and efficient selection of the most promising genotypes for further use in breeding.

First studies using the *in-vivo* DH technique to produce fixed lines from European and tropical landraces showed a generally lower testcross performances but also a high improvement potential of such materials [18], [27]. Yet, information about the *per se* performance of a broad set of DH lines derived from landraces would be of equal interest with regard to their introgression into modern breeding material. In addition to an evaluation of the phenotypic diversity captured in these lines, it would allow selection of agronomic traits relevant for line maintenance and hybrid seed production [28]. Evaluation of the molecular diversity among these DH lines and its comparison with that of the EF material could provide important information about the level of untapped genetic variation present in the landraces.

The goals of this study were to (1) evaluate the phenotypic and molecular diversity captured within DH lines derived from European landraces and compare them with the diversity of EF lines, (2) assess the breeding potential (usefulness) of DH lines derived from landraces to broaden the genetic base of the EF germplasm, and (3) compare the performance of each landrace with the synthetic population produced from their respective DH lines. The comparison of DH lines derived from the landraces *Bugard*, *Gelber Badischer* and *Schindelmeiser* with EF lines in field trials and marker assays revealed substantial phenotypic and molecular variation within the populations of DH lines, supporting their potential to broaden the genetic base of the EF germplasm.

## Results

### Experiment 1 (DH lines from landraces and elite material)

#### Mean performance reveals diversity among populations

Significant differences (*P*<0.05) were observed among the means of the populations of DH lines from landraces and the EF lines for all morphological and agronomic traits except female flowering (Table 1), as well as for all yield components (Table 2). The DH lines derived from the landrace *Bugard* (DH-BU) had on average a low emergence rate, high leaf chlorosis, low early growth rate and short anthesis-silking interval. They had short plants with intermediate ear height, well developed ear shanks and almost no husk flag leaves. The plants carried short broad ears with 10.8 kernel rows and 16.4 kernels per row on average, with a tendency to develop a second seed setting ear. The DH lines derived from the landrace *Gelber Badischer* (DH-GB) had a high emergence rate, low leaf chlorosis, high early growth rates and an intermediate anthesis-silking interval. The tall plants with high ear insertion also had long ear shanks and husk flag leaves. Their ears were long and slender, with 8.4 kernel rows and 20.3 kernels per row. The DH-GB lines showed the highest mean for 100 kernel weight among all populations. The DH lines derived from the landrace *Schindelmeiser* (DH-SC) showed values intermediate to those of DH-BU and DH-GB for most of the traits. Exceptions were ear height, ear shank and ear dry matter content, where DH-SC lines had lower means than DH-BU and DH-GB. In contrast, the DH-SC lines showed higher means for anthesis-silking interval and kernel rows. The EF lines had on average 22% higher grain yield (55.2 g plant^−1^) than the DH lines derived from landraces (43.0 g plant^−1^). Grain yield and number of kernel rows were the only traits for which the EF lines significantly (*P*<0.05) outperformed all three populations of DH lines. The founder lines EP1, F2, F7 and DK105 had a grain yield of 49.5, 38.9, 47.2 and 55.0 g plant^−1^, respectively, and were thus below the average grain yield of the EF lines.

**Table 1:** Mean, genotypic variance (*σ^2^_g_*), genotype×environment interaction variance (*σ^2^_ge_*), heritability (*h^2^*), predicted gain from selection (*ΔG*) and usefulness (*U*) at selection intensity *α* for agronomic and morphological traits of doubled haploid (DH) lines derived from the landraces *Bugard* (DH-BU, n = 36), *Gelber Badischer* (DH-GB, n = 31) and *Schindelmeiser* (DH-SC, n = 65), as well as of elite flint lines (EF, n = 106).

						α = 10%		α = 40%	
Trait	Population	Mean†	*σ^2^_g_*	*σ^2^_ge_*	*h^2^*	ΔG	*U*	ΔG	*U*
Emergence %	DH-BU	52.4a	217.72**	47.44**	0.94	25.18	77.58	13.88	66.28
	DH-GB	63.3bc	67.59**	22.95**	0.88	13.57	76.87	7.48	70.78
	DH-SC	58.8ab	134.17**	26.29**	0.93	19.66	78.46	10.84	69.64
	EF	64.8b	113.47**	42.36**	0.89	17.69	82.49	9.75	74.55
Leaf chlorosis 1–9‡	DH-BU	4.20b	0.81**	1.02**	0.76	1.38	2.82	0.76	3.44
	DH-GB	3.15a	0.46**	0.35**	0.79	1.06	2.09	0.58	2.57
	DH-SC	3.19a	0.40**	0.42**	0.74	0.96	2.23	0.53	2.66
	EF	3.10a	0.33**	0.28**	0.74	0.87	2.23	0.48	2.62
Relative growth rate GDD^−1^×10^−3^ §	DH-BU	17.2a	0.61*	0.99**	0.66	1.12	18.32	0.62	17.82
	DH-GB	18.9b	0.66**	0.00	0.84	1.31	20.21	0.72	19.62
	DH-SC	18.3b	0.91**	0.21*	0.85	1.55	19.85	0.85	19.15
	EF	17.6a	0.56**	0.68**	0.69	1.09	18.69	0.60	18.20
Female flowering GDD	DH-BU	661a	1947**	374**	0.94	75	736	41	702
	DH-GB	659a	1806**	246**	0.95	73	732	40	699
	DH-SC	656a	1386**	398**	0.92	63	719	35	691
	EF	638a	1902**	239**	0.96	75	713	41	679
Anthesis-silking interval GDD	DH-BU	31.8a	209**	126**	0.81	23	8.8	13	18.8
	DH-GB	42.3ab	430**	163**	0.88	34	8.3	19	23.3
	DH-SC	52.9b	404**	188**	0.86	33	19.9	18	34.9
	EF	34.1a	388**	83**	0.91	33	1.1	18	16.1
Plant height cm	DH-BU	134a	355**	40**	0.96	32	166	18	152
	DH-GB	164c	432**	50**	0.96	36	200	20	184
	DH-SC	144ab	329**	31**	0.96	31	175	17	161
	EF	149b	313**	16**	0.97	31	180	17	166
Ear height cm	DH-BU	49.2ab	144.94**	9.90*	0.96	20.76	69.96	11.44	60.64
	DH-GB	53.3b	63.37**	23.24**	0.86	12.99	66.29	7.16	60.46
	DH-SC	44.1a	73.95**	9.64**	0.92	14.52	58.62	8.00	52.10
	EF	51.5b	76.26**	8.10**	0.93	14.82	66.32	8.17	59.67
Ear shank 1–9‡	DH-BU	4.48b	1.38**	0.26**	0.92	1.98	2.50	1.09	3.39
	DH-GB	4.45b	1.84**	0.08	0.96	2.34	2.11	1.29	3.16
	DH-SC	3.75a	0.92**	0.38**	0.86	1.57	2.18	0.86	2.89
	EF	4.08ab	0.77**	0.06**	0.92	1.48	2.60	0.82	3.26
Husk flag leaves 1–9‡	DH-BU	1.74a	1.57**	0.00	0.96	2.16	−0.42	1.19	0.55
	DH-GB	4.19c	1.75**	1.16**	0.83	2.12	2.07	1.17	3.02
	DH-SC	3.50b	2.04**	0.94**	0.87	2.34	1.16	1.29	2.21
	EF	1.60a	0.44**	0.01	0.88	1.10	0.50	0.60	1.00

*, ** Significant at the 0.05, 0.01 probability level, respectively.

† Different letters indicate significant differences among the four populations for the respective trait.

‡ 1 = absent, 9 = pronounced.

§ Multiply the reported mean by this this value and the variance components by the square of this this value to obtain the actual numbers.

**Table 2:** Mean, genotypic variance (*σ^2^_g_*), genotype×environment interaction variance (*σ^2^_ge_*), heritability (*h^2^*), predicted gain from selection (*ΔG*) and usefulness (*U*) at selection intensity α for grain yield and yield components of doubled haploid (DH) lines derived from the landraces *Bugard* (DH-BU, n = 36), *Gelber Badischer* (DH-GB, n = 31) and *Schindelmeiser* (DH-SC, n = 65), as well as of elite flint lines (EF, n = 106).

						α = 10%		α = 40%	
Traits	Population	Mean†	*σ^2^_g_*	*σ^2^_ge_*	*h^2^*	Δ*G*	*U*	Δ*G*	*U*
Ear length cm	DH-BU	10.7a	1.54**	0.14	0.90	2.07	12.77	1.14	11.84
	DH-GB	14.1c	2.88**	1.21**	0.87	2.79	16.89	1.54	15.64
	DH-SC	12.7b	3.39**	1.05**	0.9	3.07	15.77	1.69	14.39
	EF	13.3bc	2.39**	0.29**	0.92	2.61	15.91	1.44	14.74
Ear diameter mm	DH-BU	34.3b	8.30**	1.47**	0.92	4.86	39.16	2.68	36.98
	DH-GB	30.7a	5.98**	0.48	0.92	4.13	34.83	2.28	32.98
	DH-SC	34.5b	8.75**	1.81**	0.91	4.97	39.47	2.74	37.24
	EF	33.9b	4.33**	0.25	0.91	3.49	37.39	1.93	35.83
Kernel rows #	DH-BU	10.8b	1.41**	0.11*	0.94	2.03	12.83	1.12	11.92
	DH-GB	8.4a	0.35**	0.00	0.85	0.96	9.36	0.53	8.93
	DH-SC	11.6c	1.18**	0.19**	0.91	1.82	13.42	1.01	12.61
	EF	12.9d	1.29**	0.14**	0.93	1.93	14.83	1.06	13.96
Kernels per row #	DH-BU	16.4a	6.71**	0.70	0.86	4.23	20.63	2.33	18.73
	DH-GB	20.3b	13.63**	7.20**	0.84	5.96	26.26	3.28	23.58
	DH-SC	16.4a	12.56**	3.63**	0.88	5.85	22.25	3.22	19.62
	EF	21.6b	8.07**	1.44**	0.87	4.66	26.26	2.57	24.17
Hundred kernel weight g	DH-BU	22.1a	14.20**	4.83**	0.90	6.29	28.39	3.47	25.57
	DH-GB	25.0b	6.05**	4.61**	0.79	3.85	28.85	2.12	27.12
	DH-SC	22.9a	11.46**	2.54**	0.91	5.68	28.58	3.13	26.03
	EF	21.1a	7.73**	0.72**	0.93	4.72	25.82	2.60	23.7
Ear dry matter content‡ %	DH-BU	57.4bc	20.07**	5.32**	0.93	7.60	65.00	4.19	61.59
	DH-GB	56.1ab	10.40**	5.30**	0.86	5.26	61.36	2.90	59.00
	DH-SC	53.0a	46.97**	6.49**	0.96	11.82	64.82	6.51	59.51
	EF	59.1c	11.12**	2.00**	0.94	5.69	64.79	3.14	62.24
Grain yield g plant^−1^	DH-BU	42.5a	82.68**	19.55*	0.84	14.67	57.17	8.08	50.58
	DH-GB	44.9a	65.22**	27.32*	0.79	12.63	57.53	6.96	51.86
	DH-SC	41.7a	114.47**	19.91**	0.88	17.66	59.36	9.74	51.44
	EF	55.2b	82.42**	21.61**	0.83	14.56	69.76	8.02	63.22

*, ** Significant at the 0.05, 0.01 probability level, respectively.

† Different letters indicate significant differences among the four populations for the respective trait.

‡ at 420 GDD after flowering.

Grain yield was significantly (P<0.05) associated with all yield components (Table 3), with the exception of 100 kernel weight for DH-GB. The number of kernels per row explained on average 42% of the phenotypic variation in grain yield, while ear length accounted for 23% only. Grain yield was positively associated with early growth rates of the DH-GB lines, and with plant height of the EF lines, while negative associations were observed with female flowering of the DH-SC lines and with anthesis-silking interval of the DH-GB lines. Ear dry matter content was positively associated with grain yield of the DH-GB lines, whereas the association was negative for the EF lines.

**Table 3:** Correlations of agronomic and morphologic traits with grain yield per plant within populations of doubled haploid (DH) lines derived from the landraces *Bugard* (DH-BU, n = 36), *Gelber Badischer* (DH-GB, n = 31) and *Schindelmeiser* (DH-SC, n = 65) as well as within elite flint lines (EF, n = 106).

Trait	DH-BU	DH-GB	DH-SC	EF
Emergence	0.31	0.36	−0.21	−0.20
Leaf chlorosis	−0.22	−0.23	−0.04	−0.03
Relative growth rate	0.27	0.51**	0.16	0.10
Female flowering	−0.15	−0.22	−0.33**	−0.03
Anthesis-silking interval	0.24	−0.39*	−0.17	−0.17
Plant height	0.13	0.09	0.23	0.41**
Ear height	0.33	0.07	0.1	0.18
Ear shank	0.03	−0.02	0.13	0.04
Husk flag leaves	−0.27	0.03	0.04	−0.07
Ear length	0.42*	0.41*	0.64**	0.41**
Ear diameter	0.61**	0.55**	0.58**	0.70**
Kernel rows	0.33*	0.42*	0.25*	0.45**
Kernels per row	0.69**	0.73**	0.71**	0.42**
Hundred kernel weight	0.45**	0.24	0.45**	0.42**
Ear dry matter content	−0.05	0.42*	0.23	−0.28**

*, ** Significant at the 0.05, 0.01 probability level, respectively.

**Figure 1:**
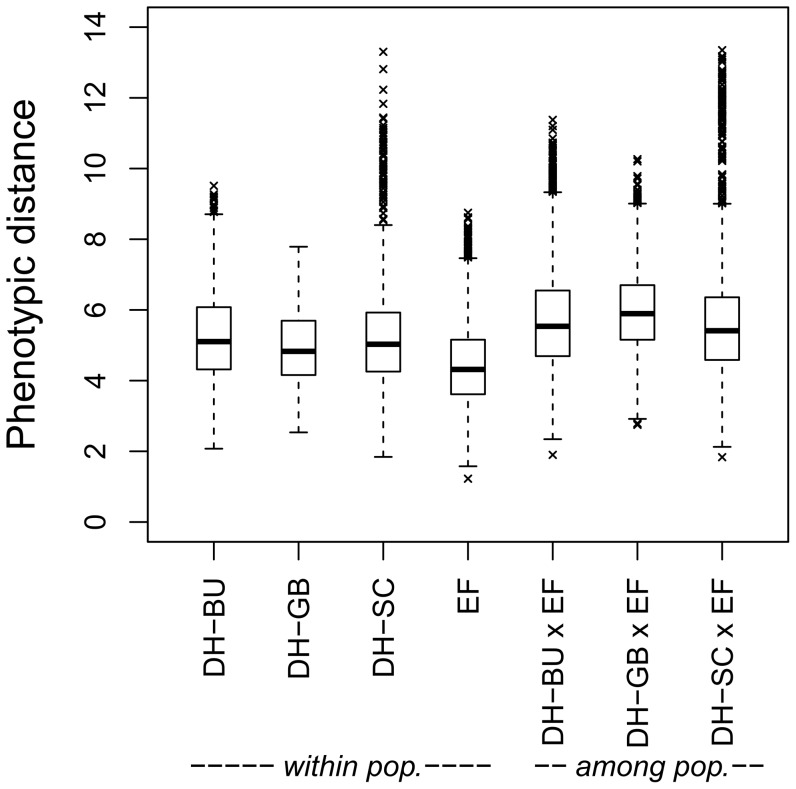
Phenotypic distances among elite flint lines and doubled haploid lines derived from landraces. Pair-wise Euclidean distances among elite flint (EF) lines and doubled haploid (DH) lines derived from the landraces *Bugard* (DH-BU), *Gelber Badischer* (DH-GB), and *Schindelmeiser* (DH-SC) were calculated from 16 morphological and agronomic traits standardized to mean zero and unit variance.

The average pair-wise phenotypic distance was 5.30 among the DH-BU lines, 4.93 among the DH-GB lines, 5.22 among the DH-SC lines, and 4.44 among the EF lines (Figure 1). Average phenotypic distance of the EF lines to the populations of DH lines was 5.72 for DH-BU, 5.97 for DH-GB, and 5.58 for DH-SC.

#### Large genotypic variances within all populations

Estimates of genotypic variance were significant (*P*<0.05) for all populations and traits (Tables 1 and 2). In most instances, the genotypic variance was higher for the populations of DH lines derived from landraces than for the EF lines. For the DH-BU lines, markedly low estimates of genotypic variance were observed for anthesis-silking interval, ear length and number of kernels per row. For the DH-GB lines, the estimates of genotypic variance were low for emergence rate, kernel rows, 100 kernel weight, ear dry matter content, and grain yield. The EF lines showed the lowest genotypic variance for leaf chlorosis, early growth rate, plant height, ear diameter, ear shank and husk flag leaves scores. Despite generally high estimates of genotype×environment interaction variance, heritabilities were moderate for growth rates of the DH-BU and EF lines (0.66 and 0.69, respectively), and high to very high (0.74 to 0.97) for the remaining traits. In most instances, the usefulness criterion at a selected fraction of α = 40% (*U_40%_*) of the populations of DH lines derived from landraces surpassed the mean of the EF lines. Important exceptions were grain yield for all populations of the DH lines and the number of kernel rows for DH-GB lines, as none of them had more than 10 kernel rows. The usefulness criterion at a selected fraction of α = 10% (*U_10%_*) of the populations of DH lines reached in many instances the usefulness of the EF lines. The top 10% DH lines (DH_10%_) selected based on index performance across all three DH line populations had an average grain yield of 54.05 g plant^−1^ while the top 10% EF lines (EF_10%_) selected according to the same index had an average grain yield of 69.43 g plant^−1^.

#### Molecular analyses reveal high genetic variation within populations

**Figure 2:**
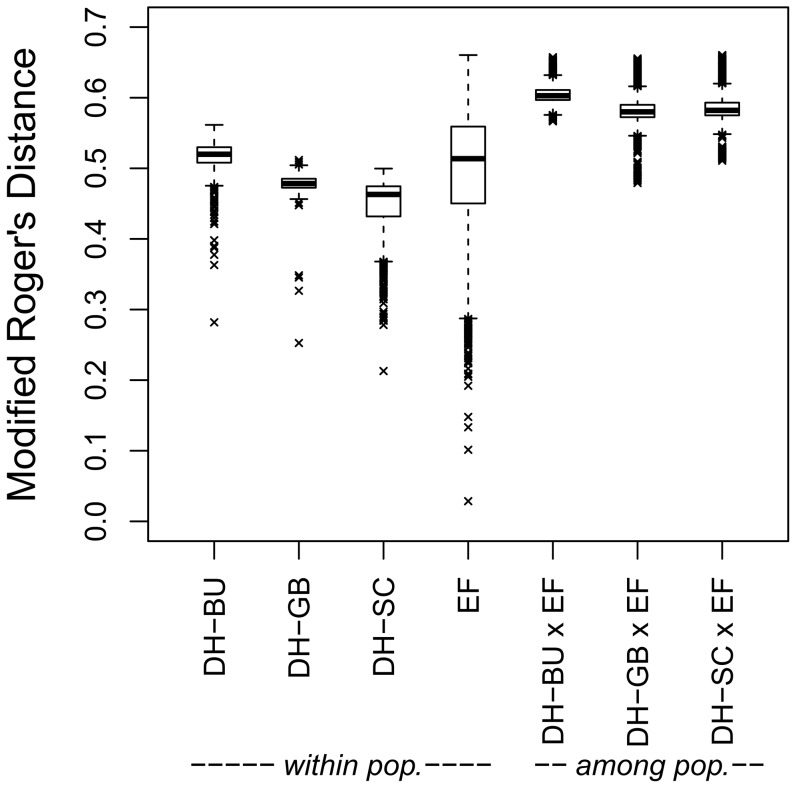
Genetic distances among elite flint lines and double haploid lines derived from landraces. Pair-wise modified Rogers' distances among elite flint (EF) lines and doubled haploid (DH) lines derived from the landraces *Bugard* (DH-BU), *Gelber Badischer* (DH-GB), and *Schindelmeiser* (DH-SC), based on 24,572 SNP markers.

Mean pair-wise modified Rogers' distance (dW) between lines calculated from 24,572 SNP markers was highest in DH-BU (0.52), and lowest in DH-SC (0.45) (Figure 2). The range of pair-wise distances was similar within the three populations of DH lines with dW values from 0.21 to 0.56. By comparison, the mean dW between the EF lines was 0.50, with values ranging from 0.03 to 0.66. Average dW between the EF lines and populations of DH lines was highest for DH-BU (0.61) and lowest for DH-GB (0.58). The minimum dW between elite lines and DH lines derived from landraces was observed between the EF line DK105 and a DH-GB line (0.48).

The number of unique alleles was highest in the EF. In total, 1,556 SNP markers were absent from the three populations of DH lines derived from landraces (Table 4), thus representing on average 16.04 unique alleles per an EF line. Among the populations of DH lines, the average number of unique alleles per line was highest (7.64) in DH-BU and lowest (0.95) in DH-SC. Nei's genetic diversity within population (*H_wi_*) was similar for the EF and DH-BU materials, whereas DH-GB and DH-SC showed lower values (Table 4). Average within population genetic diversity (*H_w_*) was 0.233 and the total genetic diversity among all flint lines (*H_t_*) was 0.312, resulting in a mean genetic differentiation (*G_ST_*) of 0.253. Linkage disequilibrium (LD) decreased below the threshold of *r* ^2^ = 0.1 within less than 0.8 Mbp in the populations of DH lines derived from landraces but only after 3.8 Mbp within the EF lines (Table 4).

**Table 4:** Number of lines (N) and population specific SNPs, genetic diversity within population (*H_wi_*), population differentiation (*G_STi_*) and extent of linkage disequilibrium (LD) within populations of doubled haploid (DH) lines derived from the landraces *Bugard* (DH-BU), *Gelber Badischer* (DH-GB) and *Schindelmeiser* (DH-SC) as well as within elite flint lines (EF).

	DH-BU	DH-GB	DH-SC	EF
**N**	36	29	60	97
**Specific SNPs**	275	87	57	1556
***H_wi_***	0.260	0.221	0.200	0.253
***G_STi_***	0.169	0.292	0.359	0.189
**LD Mbp**	0.725	0.275	0.375	3.875

### Experiment 2 (landraces and synthetic populations)

The landraces *Bugard*, *Gelber Badischer* and *Schindelmeiser* differed significantly in their mean performance (*P*<0.05) for most of the traits, with the exception of emergence rate, leaf chlorosis score, kernels per row and 100 kernel weight (Table 5). *Bugard* had the lowest early growth rate and early female flowering, and showed a narrow anthesis-silking interval. The plants were of small stature with a high ear insertion, and carried short broad ears with an average of 11.1 kernel rows and without any husk flag leaves. The high grain yield obtained with *Bugard* mainly resulted from a high number of kernels per plant. *Gelber Badischer* had a higher early growth rate, later female flowering and a wider anthesis-silking interval. The plants were tall, with similar ear height as *Bugard* and carried long slender ears with 8.7 kernel rows on average. They showed low grain yield and the lowest ear dry matter content. *Schindelmeiser* was intermediate to *Bugard* and *Gelber Badischer* for most traits. Only ear height was markedly lower than for the other two landraces.

**Table 5:** Mean performance for agronomic and morphological traits of the landraces *Bugard*, *Gelber Badischer* and *Schindelmeiser*, as well as of the corresponding synthetic populations produced by intermating the respective doubled haploid lines.

	Bugard		Gelber Badischer		Schindelmeiser		
Trait	Landrace	Synthetic	Landrace	Synthetic	Landrace	Synthetic	Synthetic effect
Emergence %	61.8a†	62.2a	62.2a	65.3a	62.0a	62.3a	
Leaf chlorosis 1–9‡	2.81ab	3.75b	2.59ab	2.53ab	2.27a	2.80ab	
Relative growth rate GDD^−1^×10^−3^ §	18.5ab	18.1a	20.0c	19.8c	19.3bc	18.9ab	
Female flowering GDD	605a	605a	675c	640b	631b	642b	**
Anthesis-silking interval GDD	44.7a	42.0a	89.5c	69.1b	68.6b	75.2b	*
Plant height cm	152a	161b	191d	176c	157ab	161b	
Ear height cm	69.8b	71.3b	74.5c	67.5b	56.0a	53.6a	**
Ear shank 1–9‡	4.0a	4.5b	4.6b	4.6b	3.8a	3.5a	
Husk flag leaves 1–9‡	1.0a	1.1a	1.9b	2.3c	1.5b	1.7b	
Ear length cm	13.5a	13.8a	17.2bc	18.9c	16.2b	15.5ab	
Ear diameter mm	39.5c	38.6c	34.7b	31.8a	35.7b	36.0b	*
Kernel rows #	11.1c	10.3b	8.7a	8.2a	12.1c	12.0c	*
Kernels per row #	26.8ab	28.0ab	30.1ab	31.8b	26.0a	26.0a	
Hundred kernel weight g	27.9b	27.0b	26.9b	26.9b	25.9ab	24.5a	
Kernel dry matter content %	66.0b	65.5b	61.7a	65.2b	65.1b	65.4b	**
Grain yield g plant^−1^	64.3b	68.2b	47.1a	48.4a	51.1a	50.3a	
Kernels per plant	227cd	252d	170a	178ab	195abc	215bc	*
Barren stalks %	2.41ab	0.00a	13.83c	7.63bc	7.47b	2.78ab	**
Common smut %	11.61a	8.41a	21.05b	13.61ab	7.34a	5.87a	*

† Different letters indicate significant differences among populations for the respective trait.

‡ 1 = absent, 9 = pronounced.

§ Multiply the reported mean by this value to obtain the actual numbers.

In the comparison of landraces with their corresponding synthetic population, significant (*P*<0.05) and systematic differences were observed for female flowering, anthesis-silking interval, ear height, ear diameter, number of kernel rows, kernel dry matter content, number of kernels per plant, number of barren stalks and incidence of common smut (Table 5). No improvement of grain yield was observed in the synthetic populations compared to the source landraces. Individual synthetic populations differed significantly (*P*<0.05) from their respective source landraces for plant height, ear shank and husk flag leaves score. Estimates of residual variance among individuals decreased significantly (*P*<0.05) in the synthetic population produced from the DH-BU lines compared to *Bugard* for plant height. Similarly, the same trend was observed in the synthetic population produced from the DH-GB lines compared to *Gelber Badischer* for number of kernel rows. Increased estimates of residual variance among individuals were observed for husk flag leaves in all synthetics (data not shown). No significant differences between estimates of residual variance among individuals were observed for the remaining traits.

## Discussion

### Recovering the diversity of landraces for breeding

Unlocking the genetic diversity present in the huge collections of maize landraces accessions by developing homozygous lines from these highly heterogeneous populations [18], [27] is of great interest for breeding. Both the single seed descent (SSD) and the *in vivo* double haploid (DH) techniques would be appropriate methods to produce fixed lines and purge the genetic load present in landraces. According to a roughly ten-fold lower efficiency of DH production when landraces are used as source population in comparison to elite crosses (Schipprack, personal communication), and following the reports on the development of first cycle inbred lines [29], high losses (∼90%) of lines due to the genetic load can be expected during the selfing or DH production process. Thus, to produce a sufficient number of unrelated inbred lines, huge numbers of individuals are needed from each population to start line development. While it is rather easy to pollinate large numbers of plants with inducers in isolation fields for a large scale DH production, producing 150 inbred lines by SSD would require selfing of around 1,500 individual plants for at least six generations. In addition, since heterozygous plants are more vigorous and as such preferred for SSD line derivation, it might even require up to eight selfing generations to produce lines with an acceptable level of homozygosity [30]. Given that only one kernel per ear is used for the next generation, approximately 12,000 hand pollinations would be required for the whole process. In comparison, assuming that 20% of the viable haploid plants produce seeds [23], only ∼3,000 hand pollinations would be required for line development (1 ear) and multiplication (3 ears) when using the DH technique. Even though chromosome doubling and selfing require additional efforts, this still results in considerably less work. Owing to the higher expected costs of SSD line development, we only used the DH technique to derive lines from the landraces, and could not compare these two methods with regard to their effect on the genetic diversity recovered.

Legitimate concerns on the selective neutrality of the *in vivo* DH technique could be raised because of the segregation distortion observed in DH lines produced by *in vitro* anther culture [31]. However, first studies on DH lines derived from single crosses of elite lines by the *in vivo* maternal DH production technique showed no systematic effect on phenotypic and allelic distribution [32], [33]. We could not monitor potential changes in the allele frequencies caused by the DH technique because the landraces themselves were not genotyped. However, to evaluate potential losses in genetic diversity, we compared the landraces with synthetic populations produced by intermating DH lines derived from the respective landrace in field trials. No bottleneck could be observed, as the residual variance among individuals was generally similar in the synthetic populations and landraces despite significant differences between mean performances for some traits (Table 5). Small but inconsistent differences in the means between the synthetic populations and the landraces (*e.g.*, plant height, husk flag leaves) might reflect random drift due to the small number of DH lines (DH-BU and DH-GB) extracted from landraces. In contrast, systematic effects (*e.g.*, female flowering, kernels per plant, number of barren stalks) suggested loss of specific alleles during the DH production or by selection during line multiplication.

Although improved seed set, reduced number of barren stalks, reduced anthesis-silking interval and lower incidence of common smut might suggest some positive purging of detrimental alleles [34], [35], the absence of grain yield improvement in the synthetic populations was disappointing and contrary to expectations [25], [27]. It must be noted, however, that seeds of the synthetic populations were produced on less vigorous homozygous lines while the seeds of the landraces were produced on vigorous heterozygous plants. It remains unclear whether purging of recessive alleles had no effect on grain yield of the resynthesized populations or whether this comparison was confounded by maternal effects [36]. Intermating each synthetic population for one generation and a comparison between the Syn-2 generation and the corresponding landrace would be necessary to resolve this issue.

### Large diversity in DH lines from landraces in comparison with EF lines

As indicated by the estimates of genotypic variances (Tables 1 and 2) and *H_wi_* (Table 4), both the phenotypic and molecular diversity within the three populations of DH lines derived from landraces were comparable to that of the EF lines. Having in mind both a high *G_ST_* of the DH-GB and DH-SC, and the fact that the EF germplasm is a mixture of various European and Northern-American materials, we can conclude that the diversity recovered from individual landraces was substantial. The *H_wi_* values from the populations of DH lines were even similar to those obtained for much larger commercial breeding populations [37]. Considering that estimates of *H_wi_* obtained from SNP marker data are approximately half than those obtained with simple sequence repeat (SSR) markers [37], [38], the *H_wi_* of the populations of DH lines were comparable to values reported for European landraces in previous studies [7], [8]. This suggests no noticeable loss of molecular diversity during the production of DH lines.

Most striking was the narrow range of *d_w_* values within the populations of DH lines compared to the one observed within the EF material (Figure 2). Except for a few pairs of lines showing *d_w_* values at half of the population mean, most likely because they originated from the same female plant in the induction crosses, all DH lines were nearly equally related. This underlines the very low population structure within the landraces in comparison with that of the EF material, suggesting that the effective population size *N_e_* of the landraces was much higher than the number of lines extracted, as re-sampling of gametes was negligible. Moreover, it supports a random sampling of gametes during DH line extraction, and underlines the value of the DH method to extract large numbers of unrelated lines from landraces.

The strongest phenotypic difference between the EF lines and the populations of DH lines derived from landraces was observed for grain yield (Table 2). With on average 22% less grain yield, the gap between the unselected DH lines and the EF lines was substantial and similar to values obtained when testcrosses of DH lines from *Gelber Badischer* and *Schindelmeiser* (22 to 26% less) were compared with commercial hybrids [18]. Grain yield of the DH lines was more tightly associated with the number of kernels per row than with ear length (Table 3). Comparison between the DH-SC and EF with similar ear size pointed to poor seed set as the main cause of the reduced grain yield of the DH lines. Trends for reduced anthesis-silking intervals, shorter husk flag leaves as well as larger ears with better seed set in modern vs. old lines (Table 1 and 2) were also reported for parental inbred lines of U.S. hybrids released between 1930 and 2000 [39]. This presumably reflects a correlated response of modern breeding germplasm to selection for grain yield under higher planting densities and led to the conclusion that the selection of lines with superior *per se* performance under stress conditions will also result in higher-yielding hybrids [40], [41].

### Potential of DH lines from landraces for broadening the genetic base of the elite flint pool

The usefulness criterion *U*(*α*) showed that the generally lower performance level of the populations of DH lines was largely compensated by large estimates of genotypic variance (Tables 1 and 2). Thus, detrimental agronomic properties (*e.g.*, lodging, poor seed set) present in the landraces can be removed prior to introgression into EF matrerials by selecting superior lines among the DH lines derived from landraces. Moreover, the yield gap can be substantially reduced because the top DH_10%_ lines reached nearly the mean grain yield performance of the EF lines. Similarly, a selection of the best performing DH lines reduced the yield gap between commercial hybrids and testcrosses of DH lines derived from *Gelber Badischer* and *Schindelmeiser* by 50% [18]. However, crosses of DH_10%_ lines with EF will still result in lower means of the progenies than crosses between top EF_10%_ lines, because the grain yield of the selected DH_10%_ lines remained far below the performance of the top EF_10%_. Yet, the usefulness of crosses does not only depend on the mean but also on the variance among their offspring. The higher phenotypic and genetic distances observed between DH lines derived from landraces and the EF lines, on one hand, and those obtained among the EF lines (Figure 1 and 2), on the other, suggest that the progeny of crosses between DH lines derived from landraces and the EF lines might release enhanced genotypic variance compared to crosses within EF [42], [43]. Previous studies confirmed the high usefulness of crosses between elite material and unselected landraces [3] or exotic material [44], and support our optimism that introgression of selected DH lines would allow broadening the genetic base of the EF material without compromising on the performance level.

We had expected that landraces would harbor numerous alleles absent from the EF germplasm. Surprisingly, we identified most of specific alleles within the EF. This might result from recent introgressions of Lancaster Sure Crop germplasm into the EF material of the University of Hohenheim (W. Schipprack, personal communication). Second, we also need to take into account some ascertainment bias of the SNP chip towards higher diversity among Lancaster germplasm than among European flint, even after the exclusion of the marker set developed by Syngenta [38]. In addition, the number of population-specific alleles might be underestimated with SNP markers because their biallelic nature will not reveal all allelic variants of a gene. It might be more appropriate to investigate the allelic variation of genes by studying haplotypes. Actually, in agreement with a high degree of recombination within the open-pollinated landraces, the rapid decrease of LD observed in the DH lines derived from landraces in comparison to the EF lines (Table 4) supports a high number of new haplotypes within the DH lines. While the introgression of new haplotypes in elite material disrupts gene combinations with positive epistatic effects [45], it also breaks negative trait associations due to linkage [46] and releases new genotypic variation. Further, the rapid decay of LD together with high genotypic variances and absence of population structure within the populations of DH lines derived from landraces enables high resolution association mapping in such germplasm. Thus, new genes and alleles of agronomic interest might be identified with high precision in the DH lines derived from landraces prior to marker-assisted introgression into the elite material.

## Conclusions

Owing to the large estimates of genotypic variance among the DH lines derived from landraces, individual lines with superior performance for agronomic and morphological traits can be selected and introgressed into the elite material. As suggested by the high phenotypic and genetic distance between the DH lines and the EF lines, the generally lower grain yield and testcross performance of DH lines derived from landraces might be well compensated by a large genotypic variance for these trait in the progenies of crosses with EF lines [3]. Further, the improvement of seed set and other traits related to fitness in the synthetic populations suggest that the DH technique might help in purging detrimental alleles present in landraces, apparently without strongly affecting the phenotypic diversity. Creation of DH lines from landraces shows great promise to broaden and improve the genetic basis of the EF breeding material without necessarily introducing negative agronomic features present in the landraces. Furthermore, the rapid decay of LD together with the high genotypic variances and absence of population structure within the populations of DH lines derived from landraces make these lines an ideal tool for high resolution association mapping.

## Materials and Methods

### Plant material

A set of 132 DH lines was produced by KWS SAAT AG (Einbeck, Germany) from the European maize landraces *Bugard* (DH-BU, n = 36), *Gelber Badischer* (DH-GB, n = 31), and *Schindelmeiser* (DH-SC, n = 65) by a proprietary *in-vivo* haploid induction technique similar to the one described by Röber et al. [23]. Passport and primary descriptors of these landraces can be found in the European Union Maize Landrace Data Base [47]. The landraces *Bugard*, *Gelber Badischer*, and *Schindelmeiser* were maintained by KWS SAAT AG. Testcross performance of the DH lines derived from the landraces *Gelber Badischer* and *Schindelmeiser* were already reported in a previous study [18]. For comparison of the DH lines from landraces with advanced European breeding material, we evaluated 106 elite flint (EF) inbred lines from the breeding program of the University of Hohenheim and an additional set of 150 elite lines (EL) belonging to other germplasm pools (Table S1). The EL material was not further analyzed in this study except for the statistical analyses of lattice designs. The most important founder lines of the European flint germplasm, *i.e.*, F2, F7, DK105 and EP1 were included in the EF material. Lines F2 and F7 are derived from the French landrace *Lacaune*, DK105 from the German landrace *Gelber Badischer* and EP1 from the Spanish landrace *Lizargarote* [5].

The landraces *Bugard*, *Gelber Badischer* and *Schindelmeiser* were multiplied in the winter nursery in Chile during the season 2009/2010 by sowing 200 plants and pollinating each row with bulked pollen from the other rows. Synthetic populations of *Bugard* (SYN-BU), *Gelber Badischer* (SYN-GB) and *Schindelmeiser* (SYN-SC) were produced by intermating all DH lines of the respective population. For this purpose, pollen of five (SYN-BU, SYN-GB) or ten (SYN-SC) DH lines was bulked to pollinate one ear of each of the remaining DH lines from the respective landrace. This procedure was repeated until all lines were used once in a pollen bulk. Equal numbers of seeds per pollinated ear were bulked to ensure an equal contribution of each DH line to the synthetics.

### Marker assays and their biometric analyses

Genomic DNA from the inbred lines was extracted from pooled leaf tissue samples of five seedlings per genotype using the CTAB method [48]. Each line was genotyped for 56,110 SNPs using the MaizeSNP50 BeadChip (Illumina Inc., San Diego, USA) [49]. Quality control of the SNP marker data was performed according to Strigens et al. [50]. Inbred lines showing more than 2% of heterozygous loci were excluded. Lines and SNP markers with call rates below 0.95, as well as SNP markers with minor allele frequency (MAF) below 5% were excluded from further analysis. To avoid ascertainment bias arising from a subset of SNP marker designed to maximize genetic distances among Stiff-stalk and non-Stiff-stalk material, we further excluded the set of 14,810 SNPs developed by Syngenta [38], [49]. In conclusion, a set of 125 DH lines derived from landraces, a set of 97 EF lines, and 24,572 SNP markers remained for genetic analyses after the quality check.

Number of population-specific SNPs and pair-wise modified Rogers' distances (*d_W_*) [51] were calculated for both the EF lines and DH lines derived from each landrace. Minimum, maximum and average *d_W_* were determined among the lines of the four groups, as well as between the EF lines and individual populations of the DH lines. Nei's total genetic diversity (*H_t_*) was estimated over all loci and lines [52]. Genetic diversity within populations (*H_wi_*) was computed for each individual population and averaged to obtain the mean genetic diversity within populations (*H_w_*). Overall genetic differentiation (*G_ST_*) was calculated as 1-(*H_w_*/*H_t_*), and population-wise genetic differentiation (*G_STi_*) as 1-(*H_wi_*/*H_t_*). Linkage disequilibrium (LD) was calculated within each population as *r* ^2^ values between all pairs of loci for each chromosome [53]. To characterize the extent of LD in Mbp within each group, *r* ^2^ values were binned according to the distance between markers in steps of 0.05 Mbp and averaged over chromosomes. The threshold of *r* ^2^ below which LD was considered non-significant was set to 0.1 [54].

### Experiment 1 (DH from landraces and elite lines)

The 256 elite inbred lines and 132 DH lines derived from landraces were divided into two sets of 200 entries each. Twelve inbred lines were common to both sets to allow for a combined analysis and adjust for potential differences between the experimental sets. The two sets were evaluated in separate but adjacent field trials laid out as a 20-by-10 alpha design with two replications [55]. Single-row plots of 3 m length with 0.75 m distance between rows were overplanted and later thinned to a final plant density of 10 plants m^−2^. The trials were conducted in 2010 in five environments in South Germany, contrasting in mean air temperature, altitude, nitrogen supply, and cultivation practice. Eckartsweier, located at an altitude of 141 m a.s.l. in the upper Rhine Valley, with the highest average temperatures (9.9°C), is considered optimal for maize cultivation, whereas Oberer Lindenhof, located at an altitude of 700 m a.s.l. on the Swabian Alb, is a marginal environment for maize growing due to low average temperature (6.6°C). The plants at Oberer Lindenhof were thus harvested already at the eight-leaf stage. Both locations were amended with fertilizer according to usual cultivation practice (150 kg N ha^−1^). In Hohenheim (400 m a.s.l., 8.8°C), the trials were conducted on a conventionally fertilized field (150 kg N ha^−1^) and on a nitrogen deficient one (0 kg N ha^−1^), where only P and K fertilization was kept at optimum. The location Kleinhohenheim (435 m.a.s.l., 8.8°C), adjacent to Hohenheim, was cultivated according to organic farming directives and amended with 50 Mg ha^−1^ organic manure of undetermined N-availability.

Sixteen traits were evaluated on a plot basis for all lines. Emergence was determined as the ratio in percent of emerged plants to sown seeds per plot before thinning. Leaf chlorosis was scored between the four-leaf and six-leaf stage on a 1 (no chlorosis) to 9 (severe chlorosis) scale. Fresh above-ground biomass in g m^−2^ was determined two to four times, depending on the location, between the four-leaf and eight-leaf stage by a non-destructive phenotyping platform described in detail by Montes et al. [56]. Relative growth rates per growing degree days (GDD) were calculated by fitting an exponential growth function to the measured fresh above-ground biomass as described in detail by Strigens et al. [57]. For calculation of GDD, minimal and maximal daily temperatures were obtained from weather stations adjacent to the field trials and base temperature was set to 10°C. Female and male flowering were determined as the GDD from sowing until silk emergence and pollen shedding in more than 50% of the plants, respectively. The anthesis-silking interval was expressed in GDD as the difference between female and male flowering. Plant and ear height in cm were determined at maturity as the approximate distance from the soil to the lowest tassel branch, by placing a level staff in the center of each plot. Ear shank and husk flag leaves were scored on a scale from 1 (absent) to 9 (very pronounced). At physiological maturity (black layer), the ears of five plants from the center of each plot were harvested by hand. To determine ear dry matter concentration, the ears were weighed before and after drying at 60°C to a constant weight. Ear length in cm and ear diameter in mm, number of kernel rows and kernels per row of the primary ear were recorded prior to shelling. Average grain yield in g plant^−1^ and 100 kernel weight in g were determined from bulked seeds of the primary and secondary ears (as far as present) of the five plants.

### Experiment 2 (Landraces and synthetic populations)

The three landraces *Bugard*, *Gelber Badischer* and *Schindelmeiser* and the corresponding three synthetic populations produced from the intermated DH lines were evaluated in a randomized complete block design with three replications. The trials were conducted in fields adjacent to Experiment 1 in the same environments except Oberer Lindenhof. Four-row plots of 3 m (Eckartsweier, Hohenheim) or 4 m (Kleinhohenheim) length with 0.75 m between rows were overplanted and later thinned to a final plant density of 9 plants m^−2^.

Emergence, leaf chlorosis, relative growth rate, female and male flowering, and anthesis-silking interval were determined on a plot basis as in Experiment 1. Plant and ear height, ear shank and husk flag leaves scores were determined on 10 plants from each of the two center rows. Occurrence of common smut (*Ustilago maydis*) and barren stalks was recorded on these 20 plants. The two center rows were harvested at maturity with a combine to determine fresh grain yield. A grain sample of ∼500 g was dried at 60°C to a constant weight to determine kernel dry matter content and 100 kernel weight. Grain yield was calculated for a final dry matter content of 85%. Five ears each were harvested by hand from the center of the two remaining outer rows, to measure ear length and diameter, kernel rows, and number of kernels per row.

### Statistical analyses

For analysis of the phenotypic data, DH-BU lines, DH-GB lines, DH-SC lines, EF lines and EL lines were considered as five populations. The following model was employed to estimate variance components in Experiment 1:
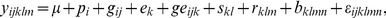
(1)where *μ* is the overall mean, *p_i_* the effect of population *i*, *g_ij_* the effect of inbred line *j* within population *i*, *e_k_* is the effect of environment *k*, *ge_ijk_* the interaction between inbred line *j* within population *i* and environment *k*, *s_kl_* the effect of set *l* within environment *k*, *r_klm_* the effect of replication *m* within trial *l*, *b_klmn_* the effect of incomplete block *n* within replication *m*, and *ε_ijklmn_* the residual. All effects in Eq. (1) except *μ* and *p_i_* were considered as random. For ear dry matter content, the sum of GDD from female flowering to harvest was additionally taken as covariate to adjust for different harvest dates. Estimates of the genotypic variance and the variance of genotype×environment interactions were computed within each population by restricted maximum likelihood, using a diagonal variance-covariance structure. Significance of the variance components was determined with the Z-test, assuming normal distribution of variance component estimates. Heterogeneity of residual variance among environments was taken into account and the pooled residual variance was calculated as the average of the individual estimates. Heritabilities (*h^2^*) were calculated within populations on an entry-mean basis, according to Hallauer et al. [30].

For calculation of the adjusted means of the lines, best linear unbiased estimates (BLUEs) were computed by considering *μ*, *p_i_*, and *g_ij_* as fixed effects in Eq. (1) while the remaining effects were considered as random. Differences between populations were tested by using Tukey's honest square difference for unbalanced data sets. To compare the performance of DH and EF lines not only for means, we estimated the predicted response from selection, Δ*G*(*α*), as well as the usefulness criterion [58], *U*(*α*), of the populations of DH and elite lines. The parameter *U*(*α*) combines the estimate of the population mean and Δ*G*(*α*) and allows a comparison between populations, with regard to the prospects to identify individuals or lines with superior performance. The parameters Δ*G*(*α*) and *U*(*α*) were computed following Prigge et al. [27] for a selected proportion of *α* = 10% (*U_10%_*) and 40% (*U_40%_*), corresponding to a selection intensity of *i* = 1.76 and 0.97, respectively. Response from selection was computed as Δ*G*(*α*) = *i*(*α*)*hσ_g_*, where *i*(*α*) is the selection intensity, *h* the square root of the heritability, and *σ_g_* the genotypic standard deviation [59]. The usefulness criterion was calculated as *U*(*α*) = *μ*±Δ*G*(*α*), where *μ* is the mean of the respective set of lines. The sign of Δ*G*(*α*) was chosen depending on whether higher values of the trait expression were regarded as positive or negative. The best 10% DH lines across the three landraces (DH_10%_) and EF lines (EF_10%_) were selected according to the index *IP* = 2×ear dry matter content+grain yield, commonly used by maize breeders in Central Europe. Within each breeding group, phenotypic correlations (*r_p_*) between grain yield and the remaining traits were determined as Pearson's correlation coefficient and the significance level was Bonferroni-corrected to account for multiple comparisons among populations. Pair-wise Euclidean distances (ED) were calculated among the EF inbred lines and DH lines derived from landraces from the adjusted entry means of the flint genotypes for the sixteen traits evaluated in Experiment 1, centered to mean zero and scaled to unit variance. Minimum, maximum and average of ED were calculated within each population, as well as between the EF lines and individual populations of DH lines.

In Experiment 2, the following model was used in a first step to estimate adjusted means of and test for differences between the six populations:
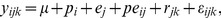
(2)where *μ* is the overall mean, *p_i_* the effect of the entry *i* (landraces and synthetic populations), *e_j_* the effect of environment *j*, *pe_ij_* the interaction between landrace or synthetic population *i* and environment *j*, *r_jk_* the effect of replication *k* within environment *j*, and *ε_ijk_* the residual. All effects except *μ* and *p_i_* were considered as random in Eq. (2). The genotypic variance within each landrace and each synthetic population was determined for traits measured on a single plant basis by estimating the residual variance among individuals within each landrace and synthetic population. An F-test was performed to evaluate the significance of differences between estimates of the residual variance among individuals. In a second step, the following model was used to test for systematic changes between source landraces and synthetic populations resulting from the use of the DH technique:
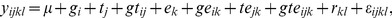
(3)where *μ* is the overall mean, *g_i_* the effect of the landrace *i* (*Bugard*, *Gelber Badischer*, *Schindelmeiser*), *t_j_* the effect of the population type *j* (source landrace vs. synthetic population), *e_k_* the effect of environment *k*, *gt_ij_*, *ge_ik_*, *te_jk_*, *and gte_ijk_* the interactions among landrace *i*, population type *j* and environment *k*, *r_kl_* the effect of replication *l* within environment *k*, and *ε_ijkl_* the residual. The parameter *μ*, *g_i_* and *t_j_* were considered as fixed in Eq. (3).

All calculations were performed within the R-environment [60]. Mixed model analyses were performed using the package ASReml for the R-environment [61].

## Supporting Information

Table S1 List of genotypes belonging to the elite flint (EF) and further elite lines (EL) populations.
(XLSX)
